# Annotations

**Published:** 1887-04-16

**Authors:** 


### ANNOTATIONS.

**Free Criticism.** An esteemed correspondent, who holds a deservedly high place in the nursing world, takes exception to our action in allowing a "Provincial Lady Superintendent" to write as she has done in reply to Miss Manson's criticisms of her former communication. Our correspondent declares this communication to be as mistaken in tone as in substance, and to be principally occupied with personal discussion of Miss Manson, instead of her paper. She goes so far as to say, indeed, that a "Provincial Lady Superintendent" has descended to the level of personal abuse. Being interested in the question of hospital management and reform, she claims to express the views of many other ladies, and adds, "Though I am willing enough to publish papers that shall be open to the fullest discussion and criticism, I cannot say that I should ever care to sign my name to anything in a Journal that would permit me to be personally attacked in my private capacity, as a retort." A reference to the paper referred to, which appears in THE HOSPITAL of the 2nd inst., and the one by "A Hospital Physician," which precedes it, will show how far our correspondent is justified in her criticism. We wish only to be fair, and to ensure that the many interests which it is our function and privilege to promote, shall receive in the columns of THE HOSPITAL a fair and impartial consideration. We cannot hold ourselves responsible for the opinions of our correspondents, who must alone be held responsible for the views they individually express; but we yield to none in our appreciation of the service rendered to the nursing profession by Miss Manson, and we hope that she, her friends, and our correspondent will believe that we would never knowingly consent to allow the private character of anyone to be attacked in these columns. Public life, especially where it is attended, as in Miss Manson's case, by useful public service, entails almost necessarily a certain amount of adverse criticism. We confess to the feeling that a good enemy in these difficult days is, as a matter of fact, about the best friend that a man or woman can have. Such an enemy is always harping on imperfections which he holds to attach to the individual criticised, and so excites a keen personal interest, resulting not infrequently in an earnest support of the opponent attacked. Besides, the world moves by differences of opinion, and honest difference is not only not discreditabl-, but positively helpful to all concerned.

**The Aberdeen Royal Infirmary.** A few weeks ago it was our duty to severely criticise the mismanagement of the City Hospital, Aberdeen. To-day we have pleasure in calling attention to the Aberdeen Royal Infirmary, which, under the able guidance of Lord Provost Henderson and Sheriff Dove Wilson, is making rapid strides towards the attainment of efficiency and economy in administration. Not content with promoting a Bill to modernise and improve the constitution of the Royal Infirmary, steps have been taken, as a fitting local memorial of Her Majesty's Jubilee, to thoroughly reconstruct the present buildings, at a cost of £30,000. A considerable part of this sum has already been subscribed, amongst the donors being five who have contributed £1,000 each, five £500 each, three £300 each, four £250 each, five £200 each, and twenty £100 each,—a truly magnificent first list of subscriptions for the inhabitants of a city with a population of little more than 100,000. Such an example must stimulate others to action and liberality; but Aberdeen and her citizens are not content to give their money and rest satisfied. They have determined to secure the best advice available, and so to provide the best guarantee that the reconstruction of the old infirmary shall not be an extended aggregation of bricks and mortar, but a genuine reconstruction, resulting in improved hygienic conditions for the treatment and care of diseases and accidents. This is a further fact worthy of remembrance, seeing the enormous sums which have been wasted in this country in the present century in so-called reconstructions of old buildings for hospital purposes.

**Amusements in Hospitals.** Last week we called attention to the great advantages which a chronic hospital, established by Mr. Jaffray at Birmingham, offered to many poor sufferers who could not be longer detained in the wards of the ordinary hospital. This aspect of the case forcibly reminds us of the value of providing amusement and relaxation for convalescent patients in hospitals. In no kind of hospital does the necessity and value of amusing occupation present itself with greater force than in the case of institutions devoted to the care of consumptive patients. The Kyrle Society has done most useful work in connection with this subject, and the thanks of the whole community are due to Her Royal Highness the Princess of Wales for her presence at the Brompton Hospital recently. "One touch of nature makes the whole world kin," and the fact that the Princess and her daughters should have devoted their talents and time to the amusement of the patients in the Brompton Hospital will be fruitful in results. It is, we believe, the first occasion upon which members of the Royal Family have so occupied themselves in this country, and if the action of the Princess of Wales and her daughters is made known throughout the kingdom, it cannot fail to arouse the affections of the people, and to stimulate the movement in favour of this new system for helping patients to relief, if not to cure. Cheerfulness in the sick is the highway to recovery, and entertainments like those given to the Brompton patients by the Princess of Wales and her daughters must, by cheering and amusing the sick, do much to promote improved health in all cases where recovery is not impossible.

